# A case of dumbbell-shaped accessory scrotum with concomitant lipoma

**DOI:** 10.1186/s40792-024-01906-w

**Published:** 2024-05-01

**Authors:** Souji Ibuka, Ryuta Saka, Hiroshi Sonobe, Ryo Tsukada, Shun Iwasaki, Rika Omote

**Affiliations:** 1 Department of Pediatric Surgery, NHO Fukuyama Medical Center, 4-14-17, Okinogami-Cho, Fukuyama, Hiroshima 720-8520 Japan; 2 Department of Diagnostic Pathology, NHO Fukuyama Medical Center, Fukuyama, Hiroshima Japan

**Keywords:** Accessory scrotum, Lipoma, Stalk, Neonate

## Abstract

**Background:**

Accessory scrotum is a congenital scrotal anomaly that is usually located anterior to the anus and frequently presents with a lipoma in a bead-like shape. Herein, we present an unusual case of an accessory scrotum with a lipoma connected by a narrow stalk and located posterior to the anus.

**Case presentation:**

A 1-month-old boy was referred to our hospital for a perineal mass present at birth. He was born at 37 weeks and 2 days, with a birth weight of 2962 g. No abnormalities occurred during the perinatal period, and the birth was uneventful. The mass had an unusual shape, comprising two masses connected by a narrow stalk. The base of the mass was posterior to the anus and was connected to the rectal mucosa. The proximal mass was elastic and soft without skinfolds, whereas the distal mass was elastic and soft with a scrotum-like skinfolds. Magnetic resonance imaging showed no spina bifida. High-intensity adipose tissues in both masses and low-intensity vessels or fibrous stroma in cord-like structures between the two masses were found on T2-weighted images. At 3 months of age, the patient underwent resection in the prone jackknife position. No tumorous lesions were connected to the mass on the rectal and coccyx sides, and the mass was completely removed, preserving the anal sphincter. Histologically, the distal mass had characteristics of a scrotum, whereas the proximal mass was exclusively a lipoma. The connecting stalk had normal skin structures and a blood vessel with parallel-running nerve bundles. The postoperative course was uneventful, and the patient was discharged on postoperative day 6.

**Conclusions:**

This case of accessory scrotum was unusual in its location and the presence of a stalk connecting the accessory scrotum and lipoma. The mechanism underlying accessory scrotum development remains unclear, and our report may impact the discourse regarding the embryological development of the accessory scrotum.

## Background

Congenital scrotal abnormalities can be classified into four types: bifid scrotum, penoscrotal transposition, ectopic scrotum, and accessory scrotum [1]. The accessory scrotum is defined as ectopic scrotal tissue accompanying a normally located and developed scrotum [2]. An accessory scrotum may arise in various perineal locations, although it is frequently found between the normal scrotum and anus [3]. While an accessory scrotum is often associated with lipoma, the concomitant lipoma is usually adjacent to accessory scrotum in a bead-like structure [3]. Herein, we report a rare case of an accessory scrotum with an unusual dumbbell-like shape, comprising a stalk between the distal accessory scrotum and proximal associated lipoma.

## Case presentation

**Fig. 1:**
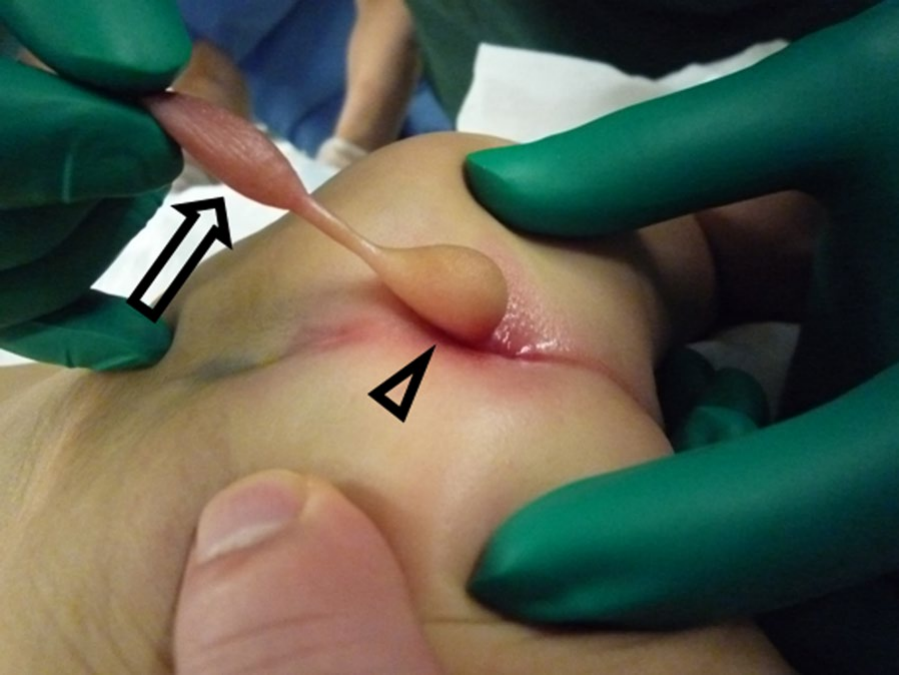
Presentation of the accessory scrotum and lipoma with the patient in the prone jackknife position. The accessory scrotum (arrow) and lipoma (arrowhead) are located posterior to the anus and connected by a stalk

**Fig. 2:**
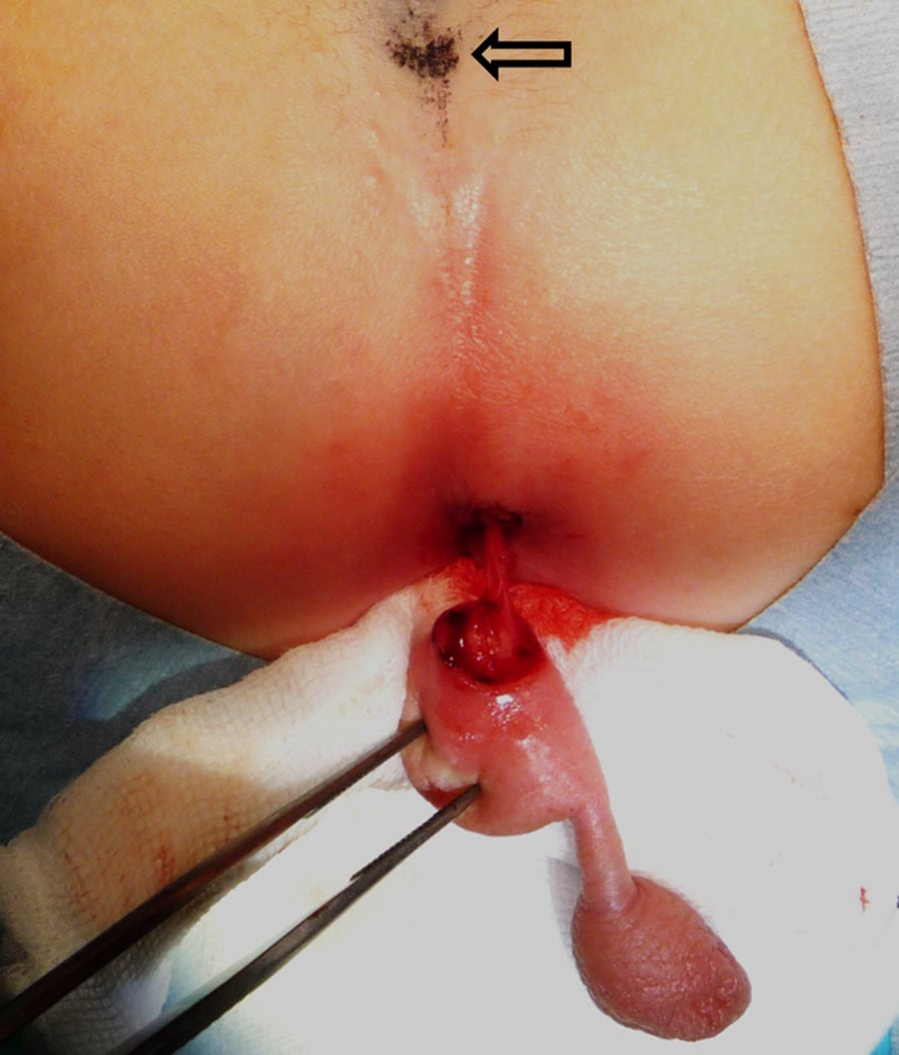
Intraoperative findings. Intraoperative photo showing that only blood vessels are connected to deep tissue and not to the coccyx (arrow). The vessels were ligated, and the accessory scrotum and lipoma were completely removed

A 1-month-old boy was referred to our hospital for a perineal mass present at birth. Delivery occurred at 37 weeks and 2 days gestation, with a birth weight of 2962 g; a selective cesarean section was performed because of a previous cesarean section. Prenatal ultrasound revealed no abnormalities, and the birth was uneventful. The patient was otherwise asymptomatic, with normal growth, development, extremity movements, urination, and defecation. The dorsal area around the coccyx was normal, without a dimple. The mass was located posterior to the anus, at the 6 o’clock position. The mass had an unusual shape, comprising two masses connected by a narrow stalk; both masses were elastic and soft, and the proximal mass had normal skin, whereas the distal mass had scrotum-like skinfolds (Fig. 1). Magnetic resonance imaging (MRI) ruled out spina bifida and revealed that both masses were composed of adipose tissues; the stalk consisted of vessels and fibrous stroma. The MRI also showed no direct anatomical relationship between the lipoma and surrounding organs, including the anal sphincter. At 3 months of age, the patient underwent surgery, with the preoperative diagnosis of an accessory scrotum with lipoma. He was placed in the jackknife position. There was no continuity to deep tissue, and the mass was removed, preserving the anal sphincter (Fig. 2). The postoperative course was uneventful, and the patient was discharged on postoperative day 6.

**Fig. 3:**
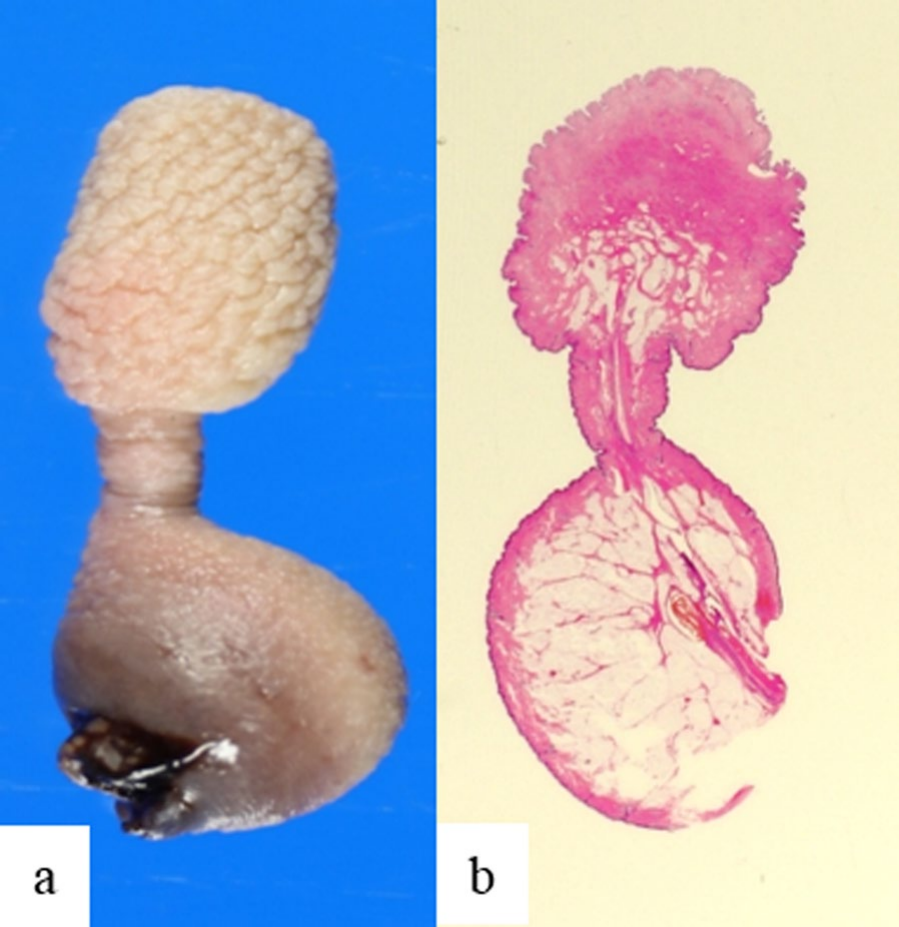
Macroscopic and histological findings. The excised and fixed lesion comprised a proximal nodule with smooth skin (lipoma) and a distal nodule with skinfolds (accessory scrotum), connected by a stalk. **a** Macroscopic features, and **b** hematoxylin and eosin stain: original magnification ×1

**Fig. 4:**
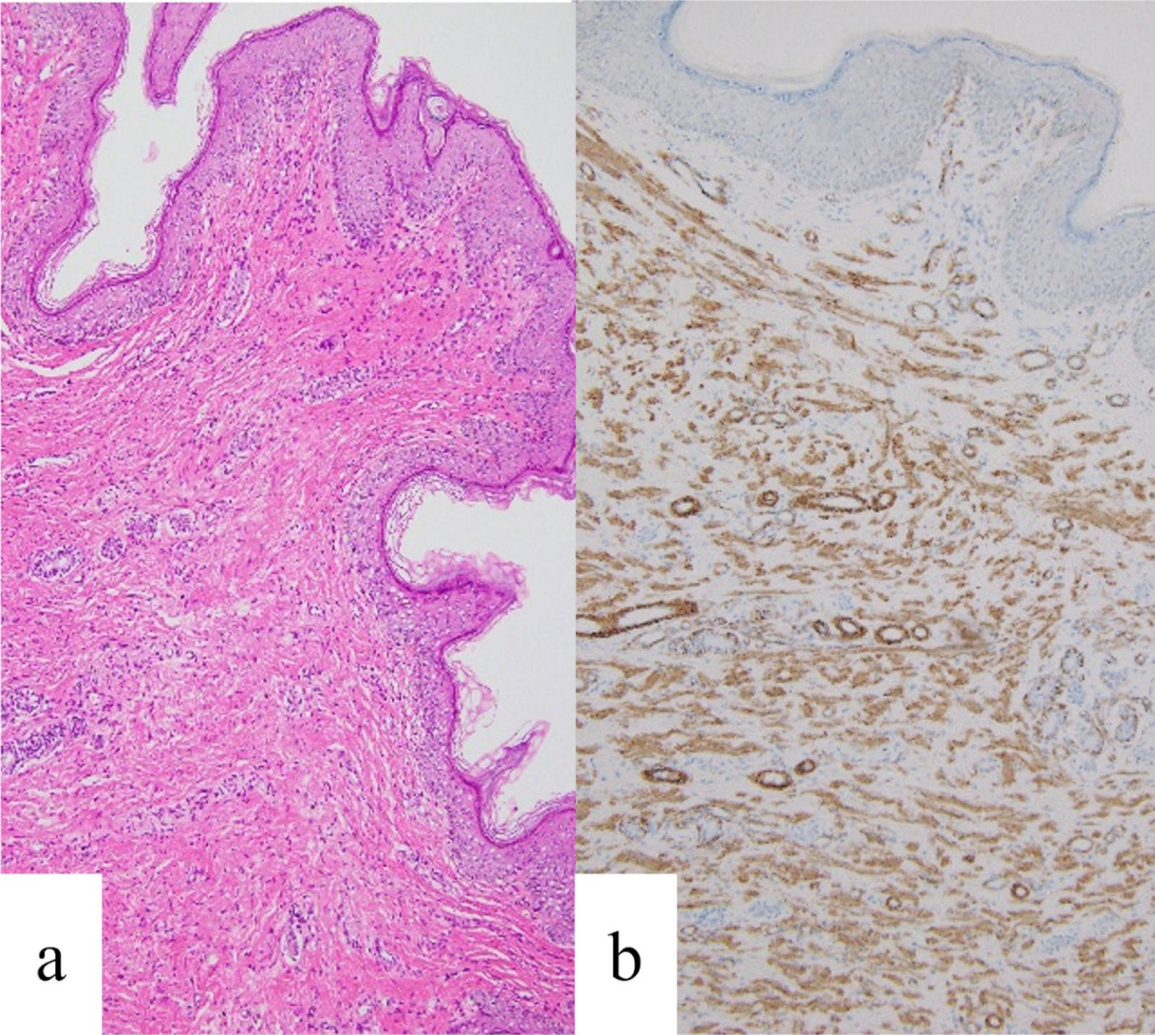
Histological findings of the distal mass (accessory scrotum). **a** Hematoxylin and eosin stain showing epidermal unevenness and the pannus carnosus composed of dense smooth muscle bundles; original magnification ×100. **b** Immunohistochemistry using anti-smooth muscle actin antibodies; original magnification ×200

**Fig. 5:**
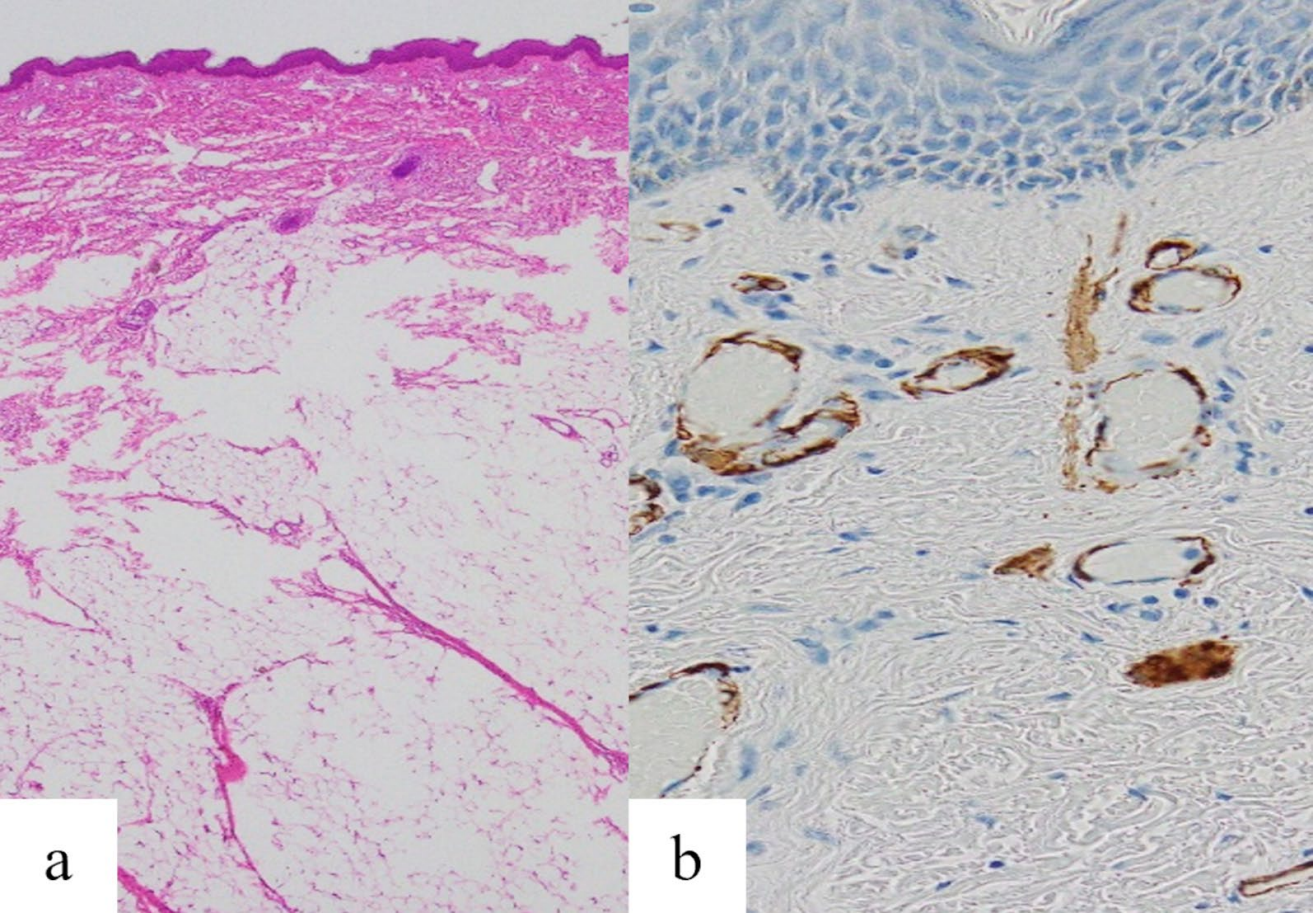
Histological findings of the proximal mass. **a** Hematoxylin and eosin staining showing the lipoma tissue; original magnification ×40. **b** Immunohistochemistry for α-smooth muscle actin showing arrector pili muscles in the dermis; original magnification ×400. The proximal mass was exclusively occupied by a lipoma, and its covering epidermis and dermis contained appendages, arrector pili muscles, and an elastic fiber network

**Fig. 6:**
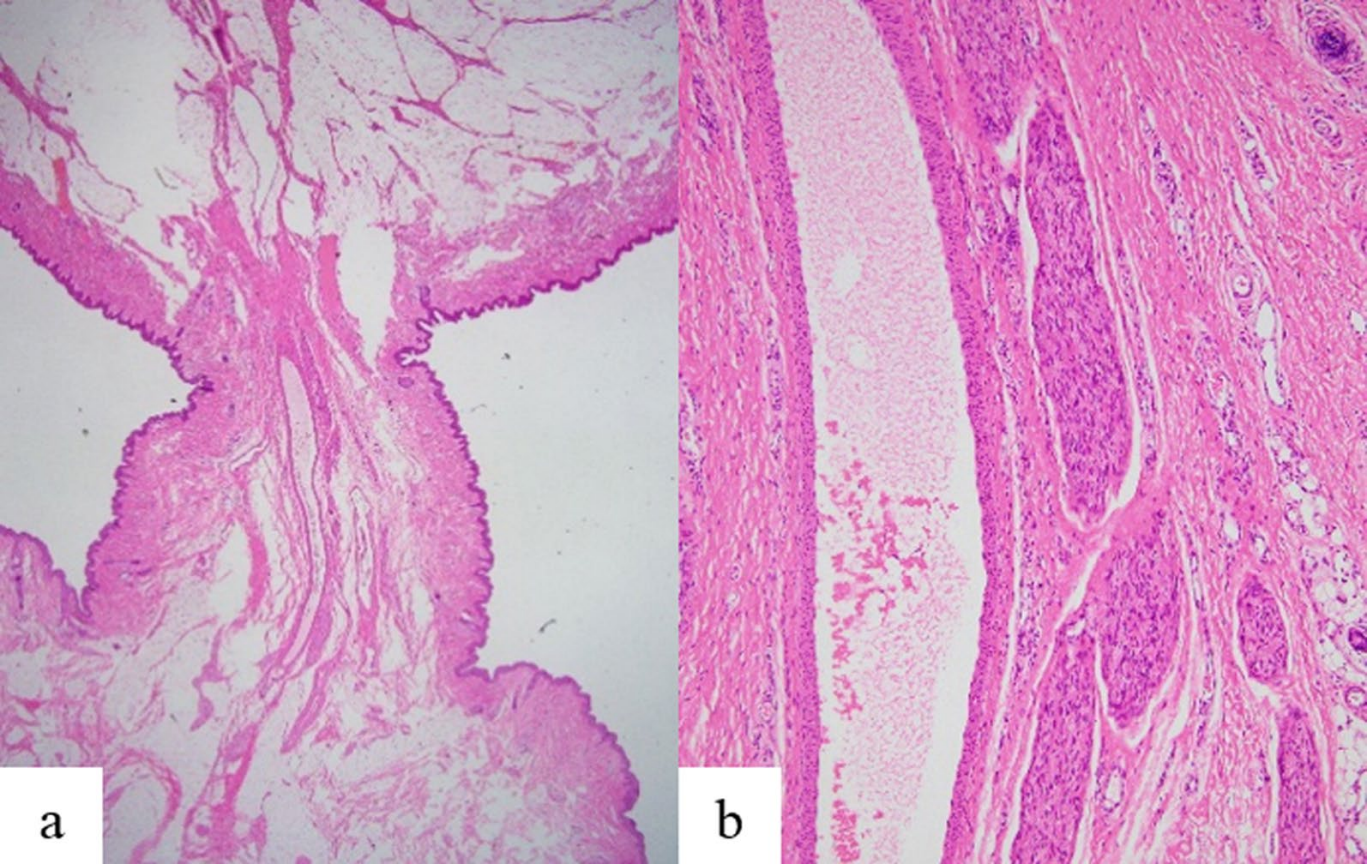
Histological findings of the stalk between the lipoma and accessory scrotum. Hematoxylin and eosin staining showing a centrally located vascular vessel and peripheral nerve bundle. **a** Original magnification ×12.5 and **b** original magnification ×200

The pathological features were as follows. Macroscopically, the mass showed a characteristic dumbbell-like shape, measuring 2.5 cm in length and comprising two masses, each approximately 1 cm in size, connected by a narrow stalk (Fig. 3). Histologically, the distal mass displayed conspicuous epidermal irregularities resembling scrotal skinfolds. In the dermis, dense and intricately running bundles were observed and were positive for α-smooth muscle antigen (α-SMA), corresponding with the tunica dartos. However, elastic fibers were not confirmed (Fig. 4). Thus, the distal mass revealed characteristic histological findings of a scrotum. The proximal mass was exclusively composed of lipoma, with normal skin (Fig. 5). The stalk between the proximal and distal masses exhibited normal skin structures, with parallel and centrally running vessels and nerve bundles (Fig. 6).

## Discussion

To date, approximately 50 case reports of accessory scrota have been published; however, the exact prevalence remains uncertain. An accessory scrotum is a scrotal anomaly where ectopic scrotal tissue develops apart from the normal scrotum [4]. In contrast with ectopic testes, the accessory scrotum does not contain testes [5]. Although accessory scrota may develop in diverse locations around the normal scrotum, these frequently occur between the normal scrotum and anus. Indeed, Ikegami et al. reviewed the localization of 41 cases of accessory scrotum published between 1980 and 2020, and only one case presented posterior to the anus [3]. In cases with concomitant lipoma, the accessory scrotum typically presents continuously with the lipoma in a bead-like shape. In our case, the proximal lipoma and distal accessory scrotum were found in an uncharacteristic dumbbell-like shape; this shape, including the presence of a stalk between an accessory scrotum and lipoma, have not previously been reported.

Macroscopic appearance is not sufficient for the diagnosis of an accessory scrotum. Definitive diagnosis is based on conspicuous epidermal irregularities resembling scrotal skinfolds and histological identification of tunica dartos. Anatomically, the scrotal tunica dartos comprises prominent smooth muscle bundles in the dermis as well as epidermal skinfolds with irregularities. In the present case, the presence of prominent smooth muscle bundles positive for α-SMA in the dermis, as well as the epidermal irregularities, supporting the diagnosis as accessory scrotum. Thus, may be helpful for the diagnosis of accessory scrotum. Furthermore, the pathological findings of the accessory scrotum and lipoma were similar to those reported previously [6]; however, only blood vessels and nerve bundles were observed in the stalk between the accessory scrotum and lipoma. This is an interesting finding with potential developmental implications. Recently, cases of prenatal diagnosis of accessory scrotum have also been reported [6, 7].

A human tail is a congenital abnormality that develops in the lumbosacral region and must be distinguished from the accessory scrotum, particularly in cases where the accessory scrotum is posterior to the anus. It is thought to arise from an irregularity in the developmental process of the vertebral portion and caudal filament that differentiate from the embryonic tail [8]. The human tail has been characterized in reports from Harrison, Dao et al., and Lin et al., although no clear definition has been established [8–10]. Apart from our case, the human tail is typically covered with normal skin and associated with tunica dartos [11]. We ultimately diagnosed an accessory scrotum in our case.

In addition to concomitant lipoma, various congenital anomalies have been reported to be associated with the accessory scrotum, including anorectal malformation, urogenital anomalies, and vertebral or bone anomalies. Reviews have shown that lipoma and anorectal malformation are most common, with a prevalence of approximately 60–80% and 18.6%, respectively [3, 6]. MRI may be useful for screening for congenital malformations associated with the accessory scrotum.

However, simple excision is generally sufficient for treating accessory scrotums, and no recurrence has been reported.

During normal scrotal development, a pair of genital swellings forms outside the genital tubercle or cloacal fold around the fourth week of gestation, and these differentiate into scrotal swellings. Subsequently, around the 12th week of gestation, the scrotal swellings move caudally from the inguinal position, each forming half of the scrotum and fusing at the scrotal raphe. The mechanism underlying the development of an accessory scrotum remains unclear, although several theories have been postulated [1, 2, 8]. For example, Lamm and Kaplan hypothesized that the labioscrotal swelling divides into two parts early in the embryonic period, becoming an accessory scrotum [2]. In our case, the accessory scrotum was located posterior to the anus, which cannot be explained by this hypothesis. While two primordia of the labioscrotal swelling arise during normal development, Takayasu et al. postulated that three develop in case of accessory scrotum [12]. According to the explanation, in our case, it should be considered that one of them developed on posterior to the anus ectopically. In addition, Sule et al. speculated that some mesenchymal tissue may transform into the lipoma, thereby causing the labioscrotal swelling to migrate away from the scrotum [1]. However, none of these hypotheses effectively explain our case in a unified way. The presence of a stalk in our case might suggest a different underlying mechanism that warrants further investigation.

## Conclusions

We report an unusual case of an accessory scrotum located posterior to the anus, with the accessory scrotum and lipoma connected by a narrow stalk. The underlying mechanism for the development of an accessory scrotum remains unclear, and additional case reports and histopathological findings are required for further elucidation. Our report provides new findings that may impact the discourse around the embryological development of the accessory scrotum.

## Data Availability

Data sharing is not applicable to this article as no datasets were generated or analyzed during the current study.

## Abbreviations

MRI: Magnetic resonance imaging
α-SMA: α-Smooth muscle antigen
